# Dr. Walter R. Francis

**Published:** 1906-01

**Authors:** 


Dr. Walter R. Francis, of Marion Ind., a graduate of the University of Buffalo, 1876, died at the home of his son, Dr. Lee Martin Francis, in Buffalo, December 28, 1905, aged 52 years...

